# Social Uses of Personal Health Information Within PatientsLikeMe, an Online Patient Community: What Can Happen When Patients Have Access to One Another’s Data

**DOI:** 10.2196/jmir.1053

**Published:** 2008-05-27

**Authors:** Jeana H Frost, Michael P Massagli

**Affiliations:** ^1^PatientsLikeMe IncCambridgeMAUSA

**Keywords:** Personal health records, data visualization, personal monitoring, technology, health care, self-help devices, personal tracking, social support, online support group, online health community

## Abstract

**Background:**

This project investigates the ways in which patients respond to the shared use of what is often considered private information: personal health data. There is a growing demand for patient access to personal health records. The predominant model for this record is a repository of all clinically relevant health information kept securely and viewed privately by patients and their health care providers. While this type of record does seem to have beneficial effects for the patient–physician relationship, the complexity and novelty of these data coupled with the lack of research in this area means the utility of personal health information for the primary stakeholders—the patients—is not well documented or understood.

**Objective:**

PatientsLikeMe is an online community built to support information exchange between patients. The site provides customized disease-specific outcome and visualization tools to help patients understand and share information about their condition. We begin this paper by describing the components and design of the online community. We then identify and analyze how users of this platform reference personal health information within patient-to-patient dialogues.

**Methods:**

Patients diagnosed with amyotrophic lateral sclerosis (ALS) post data on their current treatments, symptoms, and outcomes. These data are displayed graphically within personal health profiles and are reflected in composite community-level symptom and treatment reports. Users review and discuss these data within the Forum, private messaging, and comments posted on each other’s profiles. We analyzed member communications that referenced individual-level personal health data to determine how patient peers use personal health information within patient-to-patient exchanges.

**Results:**

Qualitative analysis of a sample of 123 comments (about 2% of the total) posted within the community revealed a variety of commenting and questioning behaviors by patient members. Members referenced data to locate others with particular experiences to answer specific health-related questions, to proffer personally acquired disease-management knowledge to those most likely to benefit from it, and to foster and solidify relationships based on shared concerns.

**Conclusions:**

Few studies examine the use of personal health information by patients themselves. This project suggests how patients who choose to explicitly share health data within a community may benefit from the process, helping them engage in dialogues that may inform disease self-management. We recommend that future designs make each patient’s health information as clear as possible, automate matching of people with similar conditions and using similar treatments, and integrate data into online platforms for health conversations.

## Introduction

This project investigates how patients react to the shared use of what is often considered private information: personal health data. Encouraged by technological trends and policies promoting patients’ rights, there is a mounting demand for flexible access to personal health information [1]. While personal health information systems vary, the predominant model is that of a central repository for *all* health information generated within clinical contexts (eg, health history, diagnoses, allergies, current treatments) that is kept securely for view only by patients and their health care providers [2].

While research in this area is still sparse, this type of record does seem to have beneficial effects for the patient–physician relationship. Provider-supplied personal health records have been shown to improve the communication and trust between the patients and health care providers [1,3] and the completeness of patient-reported data and the quality of the clinical encounter [4]. Still, the utility of a personal health information system for the primary stakeholders themselves—the patients—is not well documented or understood. One risk is that a collection of static medical information may be overly complex for the patient and therefore overwhelming. The prospect of correctly interpreting a large corpus of statically presented electronic health records causes concern even for some physicians [5]. As a result, a medical informatics working group asserted that the ideal personal health record is more than just a static repository for patient data; it should combine data, knowledge, and software tools to help patients become active participants in their own care [6].

This paper reports on a health information system, PatientsLikeMe, designed specifically for patients to use themselves and in cooperation with other patients with the same disease. In this system, patients report their relevant health information, which is presented as coherent graphical displays on their profile. Member profiles are posted where other members can have access to them, providing a basis for passive information sharing and active dialogue among patients.

This system is based on two assumptions. First, that given appropriate tools, patients will be able to interpret and learn from visual displays of personal health data [7]. This assumption is built on work on “imagery as data” in health care, suggesting that, through collecting, analyzing, and explaining visual data for themselves, patients can gauge the impact of daily behavior on health outcomes [8,9]. Second, sharing personal health data and collaboratively reviewing and critiquing it will enhance utility of the data for each contributor. Research has shown that peer-led communities that do not use personal data have documented benefits for patient knowledge, discussion, and health care utilization: users not only provide one another with support, they teach each other the science and medical information they need to understand their disease [10] and empower one another to seek out physicians who will recognize and treat their illness [11]. Communities have been shown to support reciprocal information sharing and help move participants from information gathering to positive behavioral change [12] and to provide a venue for patients to discuss morality and medical ethics [13]. Few studies isolate the effect of peer-to-peer communities on health outcomes [14]. Outside of the health domain, one quality of social Web, or Web 2.0, applications is that the applications gain value through their use [15]. Web 2.0 communities compile resources and create shared knowledge that is beyond the scope of a single individual. Framing online patient interaction around displays of personal health information can create a Web 2.0 community that may enrich patient conversations around health practice.

In this work we focus on patients who have an incurable and relatively rare life-altering disease. We do so because these patients may benefit more than other patients from a personal health record [1,2] and because their mobility constraints complicate face-to-face meetings. The platform was conceptualized for a broad set of conditions and was first implemented for amyotrophic lateral sclerosis (ALS), also called motor neuron disease or Lou Gehrig’s disease. ALS is a rare and fatal neurodegenerative disease that begins with loss of voluntary motor function and progresses to the inability to communicate, swallow, and breathe unaided. There is no cure for ALS, but there is one FDA-approved drug for its treatment, rilozule (Rilutek), which marginally lengthens life [16]. Patients use other methods to manage some of the symptoms (eg, fluoxetine [Prozac], an antidepressant, to help reduce excessive saliva) and assistive technologies to take over when biological systems fail. ALS patients and their caregivers have to decide when and if to use end-of-life interventions such as a feeding tube or ventilator.

The PatientsLikeMe platform is being continuously reviewed to understand how this model of data sharing impacts patient participation in medical decisions and organization of daily self-care practices. The primary question of the current study was how patients explicitly utilize visual displays of health information to communicate with specific patients about their treatments and disease experience. We also sought to describe the kind of dialogues that emerge when individual health information is made available within a patient community. To successfully engage in these discussions implies both the ability to draw useful conclusions from data and a level of comfort with sharing, what is often considered, personal information. We sought to answer this question by compiling and analyzing the kinds of questions, comments, and discussions that relate directly to shared, personal medical information.

## Methods

This was a design-based qualitative research study [17,18] to examine how users of the online PatientsLikeMe ALS community refer to data in discussions with specific peers. In this preliminary study, we only focus on how users employ elements of another user’s personal health profile in a discussion with that user.

### The Platform

The PatientsLikeMe ALS community was opened to the public in March 2006. Patients join the site based on the recommendation of their health providers, other patients, or patient blogs or after finding the site through online searches and Google “ad words.” A year and a half after launch, the community contained 1570 verified patients, about 1140 living in the United States. These members represent almost 4% of the estimated ALS cases in the United States [19].

### Personal Health Profiles and Data

**Figure 1:**
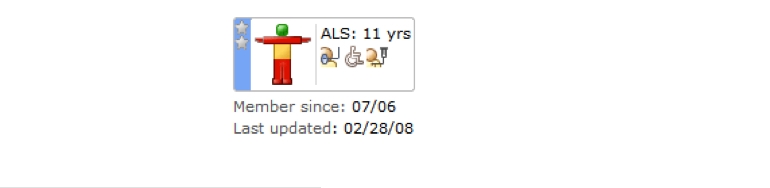
Individual summary information (the “nugget”)

**Figure 2:**
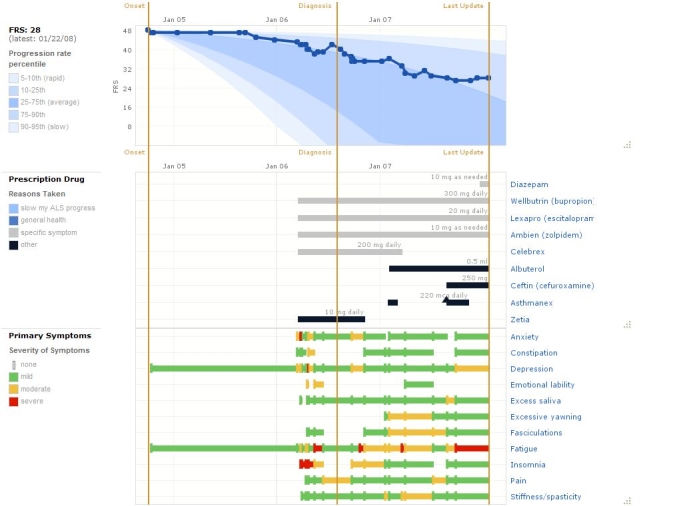
Charts comprising the personal profile

On PatientsLikeMe, each patient enters a combination of structured and unstructured data, which are compiled and presented as a profile of his or her health history and shared within the site. Profiles contain a summary representation of the patient’s current status: a diagram that maps functional impairment to areas of the body (Figure 1), a personal picture, an autobiographical statement, a diagnosis history, and a series of charts. The “nugget” summary diagram displays the current function score as a color code mapped onto affected areas of the body as well as the number of years with the disease, an iconic representation of the equipment currently used, and stars indicating level of participation on the site (see Figure 1). As in similar projects [20-22], PatientsLikeMe created a graphical display of health information as an alternative to static lists and tables in order to make the data more accessible. The primary chart on the ALS site is a line graph of the individual’s functional level over time, superimposed onto a backdrop of population-level data (Figure 2). Function is assessed through an adaptation of the clinically validated, self-administered form of the revised ALS functional rating scale (ALSFRS-R) [23].

Below the functional rating scale (FRS) chart are modified Gantt charts representing all the treatments taken and symptoms experienced by the patient. Although each user is asked about a core set of common symptoms, both treatments and additional symptoms are built with a flexible architecture such that patients define and extend the underlying ontology. In other systems, Gantt charts used to depict patient information facilitated faster comparisons between data types and improved the recall of medical information in comparison to tabular data [24]. The patient can rearrange charts within the profile to explore relationships between data types. The profile is available for personal use and to be browsed and critiqued by other users of the site.

### Aggregate Resources

Data are also aggregated from all individuals in the community to create community summaries of treatments and symptoms. Treatment reports contain standard descriptions of the treatment and display community-level data (eg, distribution of dosage levels, time on the treatment, reasons individuals have started and stopped the treatment) and relevant content culled from the Forum on users’ impressions of the treatment. Symptom reports show analogous information: the prevalence and severity in the community of each symptom and the treatments people are taking for each one. Each element in these reports is hyperlinked to related items of interest, for example, to other people taking the treatment for the same reason or in the same amount or to Forum posts on that topic.

### Social Tools

Using search and browsing tools, members can locate other patients in similar circumstances and with shared medical experiences. Members discuss the profiles and reports as well as general health concerns through the Forum, private messages, and comments they post on one another’s profiles. The Forum is a threaded dialogue available to every member of the community to pose questions, research findings, share coping strategies, and so forth. Private messages are emails sent from one user to another within the site; they are not read by other users or site administrators. Comments are remarks that one user posts on another’s profile, which are viewable by anyone in the community. Users can delete comments from their own profile. Each contribution made using any of these functions is labeled with a graphic representation (the nugget) giving a snapshot view of the contributor’s history and health status; the nugget is also linked to the user’s complete profile.

### Data Selection

On the site, there are five main categories of personal health data reported within each profile: the “about me” section (demographics, place of residence, and disease history data), a free-form biographical essay, functional ratings, treatments, and symptoms. Users interact with one another in three ways: the Forum, private messages, and comments posted to patient profiles.

For the present inquiry, we were interested in user remarks that refer to another’s individual-level personal health data. On the site, these data are displayed in the personal profile. We excluded forum posts, which are not designed to connect discussion and data of another specific user’s experience. Private messages were not analyzed because we do not access or read the content of private messages sent within the site. The analysis focused on the comments left on personal profiles. These are of prime interest in this analysis for two reasons. First, their proximity, posted at the end of the profile, may lead users to reference profile data within their comments. Second, their accessibility to all users defines them as part of the site available for research purposes.

### Sampling

Over the history of the site (December 2006 to February 2008), users in the ALS community generated a total of 17,059 comments affixed to another user’s profile. More than half of these included a predefined message—“Thank you for filling out your profile!”—that can be created with a single click, edited, and then sent. To date, 7852 user-created comments have been composed from scratch, so we focused on these original messages in the analysis. A total of 63% (986/1570) of the patients in the study period posted at least one original comment on the site. To identify comments that explicitly referenced profile data, we used a strategic sampling procedure. In a preliminary analysis of 500 original comments, we identified phrases that commonly co-occurred with references to profile data. These phrases were “I see you,” “I can see you,” and “notice you.” Approximately 30% of the 500 comments contained these phrases. No other pattern could be identified to characterize the remaining comments. An automated search of the full set of 7852 comments identified all postings that contained any of the specified phrases and added these comments to a database table for manual analysis, along with the relevant demographic data and whether these comments resulted in a response. Privacy concerns were addressed by not collecting identifying information and changing the demographic data for published segments. Using a grounded theory approach [25], a set of codes was developed. Using this set of codes, each comment was independently coded by each of the authors, differences were reconciled, and then themes were identified and discussed by both authors. To better understand how these comments fit into larger dialogues, we documented whether the comments initiated the exchange and if the commenter received responses in the form of either a private message or a comment. We tallied the number of comment and private message responses (without looking at the message contents).

## Results

We identified 123 postings by 95 users that met the criteria based on the key phrases. Among these comments, more referred to treatments (29/123, 23%) than to symptoms or outcomes (9/123, 7%). Almost half of the comments (56/123, 45.5%) included at least one question, and half of these questions were explicit requests for advice (34/123, 28%).

The following are typical examples of comments in three major categories: (1) targeted questions to others with relevant experience, (2) advice and recommendations, and (3) forming and solidifying relationships based on similarity. We also estimated how many of these comments led to ongoing discussion among users. Names of users have been changed.

### Targeted Questions to Others With a Shared Experience

When considering a new treatment, one user observed what another member was using and stated:

I notice you are using ginger root and you believe it is slowing your progression. I'm very interested in this. Can you tell me more about how it's working for you?

Another user, also curious about a nutraceutical, conducted a more complete inquiry. He sent almost identical versions of an in-depth request for information rather than addressing a specific comment to each individual on the treatment:

I see you are using Glyconutrients. What are the exact ones that you're using, how long have you been using them for, and what benefits if any have you seen. I have heard a lot of encouraging things about them, but I have yet to hear anything about their use by ALS patients. Are they helping with a particular symptom? Please let me know what you have learned by taking these supplements. Blessings to you and your family.

In such comments, users with a particular treatment question often addressed their question to other members already using that treatment. For the above two cases, the questions were about nutraceuticals and their perceived efficacy. In other comments, users asked about prescription pharmaceuticals, dosage levels, or experience using a piece of equipment. In all of these scenarios, one user with a question apparently identified another user with relevant experience and then asked about his or her perception of the treatment’s efficacy.

The graphic depiction of the length of time on a treatment (in the Gantt charts) pointed one user to identify another as an appropriate recipient for his question. Since this man was considering a feeding tube, he asked a woman on the site about her experience:

Peter [Jen], I'm a new member of PLM like yourself. I notice you have had a tube for about 8 months. I'm having difficulty eating, so the neurologist suggested I look into getting one. My meeting with the gastroenterologist did not leave me with desire to get one. It would help me if you would send me a message about your experience, pro and con, with your feeding tube.

In this case, Peter explicitly referenced the amount of time Jen had been using an assistive technology as evidence of her value as an advisor. Although he had consulted with health care providers, he sought out another patient’s opinion with the implication that it would contribute to his own decision.

In the above cases, users identified a single feature of a profile then asked an appropriate question. Other users made more sophisticated observations based on multiple charts and data types. For example, Adam, an ALS patient considering the use of a breathing assistance device—bilevel positive airway pressure (BiPAP)—asked the following:

Adam Hi [D] I am [Adam] in the PLM web site. My als was like yours breathing onset. I see your FRS improved a bit after you went onto BIPAP in april 06. Did it in fact make that much difference.??

**Figure 3:**
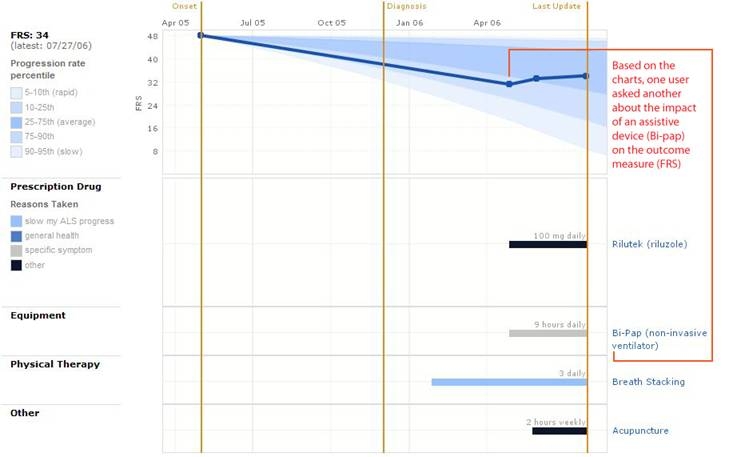
Patient profile of PatientsLikeMe member “D” (with added explanatory remark "Based on the charts..", which is not part of the original screenshot)

To ask this question, Adam apparently cross-referenced two charts in the profile to see the relationship between beginning to use various interventions, including a BiPAP, which is displayed in one type of chart, and experiencing improved function, which is displayed in another. In this case, the relationship looked clear (Figure 3). In fact, the displayed clarity of the relationship appeared to give Adam pause since he asked for confirmation.

In the preceding examples, users with a specific question identified another member and addressed their question to him or her. The criterion leading to that identification appears to be simply taking the medication or using the treatment. In one case, a user referenced the amount of time a member had been using a technology as a factor in identifying an individual as a credible resource. Using other members as a resource to inform treatment decisions emerged as a reoccurring use of comments within the site (29/123, 24%).

### Advice and Recommendations

Browsing the site, users frequently posted their remarks on one another’s profiles, in some cases sharing their own relevant experience. One man observed another’s symptom:

I see you note emotional lability. I had that very bad, but now I take a compound of dextromethorphan and quinidine that controls it beautifully.

In this case, a user offered personally acquired knowledge to another member listing a shared symptom (depicted within a modified Gantt chart). This was not an isolated instance: in five of the comments users provided similar recommendations specifically around observed symptoms, including bed sores and cramping. In each case, the comment offered advice based on a positive personal experience and included a treatment recommendation and a method for administering that treatment.

Users’ advice went beyond sharing personal treatment and symptom experiences. Within the “about me” section of the profile, many users provided their city and state of residence. Some members reading the profile referenced this information to make geographically appropriate suggestions. For example, one patient wrote to a caregiver on the site about a local support group:

Hi [Bill]. ... There will be another ALS support group starting up next Tuesday Feb.20th in Holt. Just checking to see if your parents were interested...

In similar references to location, four users either mentioned or explicitly invited others to support groups.

In one case, a user noted the individual’s region and type of onset to suggest a research study on a new technology designed to address her specific situation:

George [Joanne], I see you are legs onset; have you heard about the new diaphragm implants they are doing at Case-Western and Johns Hopkins? It means you don't have to vent to breathe.

George had a piece of information about a location- and topic-specific resource available to patients. He referenced Joanne’s diagnosis history (onset type) to make a recommendation. Using posted data, he was able to connect the individual to the resource.

In these cases, users offered advice and recommendations to others. In most cases, these recommendations stemmed from personal experience with taking a drug or using a device, but they also stemmed from personal research, as in the last example where a member offered knowledge of a research study to another user. In other online applications, individuals may share personal experience through messages broadcasted to a large audience. In this context, users delivered targeted messages to particular users they think may benefit from them.

### Forming and Solidifying Relationships Based on Similarity

Comments also function as a mechanism for creating and maintaining relationships, particularly around points of similarity: 25% (31/123) of the comments we analyzed identified a shared attribute, hobby, or concern within a broader comment or question. Locating a similar patient, one member quoted what they had in common medically as a basis to invite further contact:

Hi [Michael], I see we are pretty similar. I am 62 dx 11/06 with leg onset. I need a walker to help me walk. I move slower and have had a few recent falls due to my leg dragging. I would like to be available if you want to compare progress. I started noticing symptoms a year ago, but just dx this month.

Referencing diagnosis history, this user made a connection with another member in the community. For patients in unusual situations, the site allows for finding a similar individual even when there are only a few. In the following case, another user expressed her pleasure in finding others with a shared but atypical disease progression:

hi [Rachel]. yes same boat indeed. i am so glad to find this site because i see there are many of us with slower progression than stereotypical. the support groups locally really focus of immediate need patients and us long timers are not so immediate except we still have concerns and fears, etc so it has been so great to see how long timers cope with losing our function slowly and wondering which part is going to fail next. hers my personal email; … id love to talk more.

In these cases, the first patient had explicitly invited further contact, and the second suggested a willingness to share data even beyond the anonymous structure of the site by giving her personal email address.

In addition, there were examples of patients seeking out others based on non-medical criteria: 18 of the 31 comments on similarity were based on non-medical criteria including location, employment history, astrological sign, and shared interests. As with the medically based examples, the site facilitated meeting of people with shared concerns—people who probably would not have met offline.

### Initiating Ongoing Discussion

All 123 comments were analyzed as single units, but the reality is that comments may occur within ongoing exchanges. Without looking at the contents of private messages, we examined the full exchange of comments and messages between the sender and the recipient of all the comments studied. We found that these comments served to both continue an exchange between the two users (59/123, 48%) and initiate new exchanges (64/123, 52%). In the initiate cases, 56% (36/64) of the comments received at least one reply. In more than half the replies (20/36, 55%), the recipient continued the exchange in the “public” sphere of the site either through comments only (12/36, 33%) or through a combination of private messages and comments (8/36, 22%). On the other hand, among the cases where a comment emerged in an ongoing exchange, 57 of 59 comments were responded to, with comments being used in 68% of the exchanges.

## Discussion

While there is growing demand by patients for access to their own health data, there is little information on how other people will use these data if they are made available to others with similar medical concerns. For this study, we made use of a platform designed to help patients share personal health information by representing key data in a standardized graphical format within accessible personal profiles. By looking at one of the social behaviors within this platform—comments that explicitly reference other users’ health data—we begin to get a sense of how patients employ this information.

This analysis identifies and analyzes a small but illustrative subset of all user-generated comments—those in which members explicitly refer to another’s data, indicating that they have examined and interpreted posted medical information. We see cases where such data serve as a focal point for detailed discussions of health-related topics such as treatment decisions and symptom control. We identify three themes in the comments studied: asking advice of a user with a particular experience, offering advice to a user with a specific symptom or health problem, and fostering relationships based on shared attributes. In other situations, research has shown that perceived similarity to self in attributes and attitudes predicts positive social evaluations [26,27]; in these comments on PatientsLikeMe, similarity appears to operate analogously, heightening interest in another user. Unlike in other domains, this type of similarity—based specifically on shared medical characteristics—may contribute to positive medical outcomes as others in similar situations may be able to offer pertinent advice and suggestions and logistic as well as social support. Although small in number, the comments selected for this study represent an undetermined fraction of all uses of profile data. Nevertheless, they offer insight into the potential value of patients sharing health information.

This study represents a first examination of the use of shared medical information, which is still a novel model for personal health data. It is limited in scope by several factors, including the functionality studied and the sampling method employed. In this study, we focused deliberately on posted comments and then only on those that fit a predefined search criterion for identifying comments likely to explicitly reference another user’s health data. Our sample is only a small percentage of the total number of elements on the multidimensional site. As a result, we do not know what an analysis of all the references to data on the site would reveal. For example, data may function to define the history of a patient, which in turn enhances the forum conversation; viewing another’s profile may reduce a sense of isolation that could result from living with a disease; and other profiles may help individuals contextualize their own experience within a community of fellow patients. Future research based on interviews and surveys could investigate these possibilities more thoroughly. We also need to understand why the comments that passed our screen for prima facie use of data are only a small percentage of the total comments generated on the site. Perhaps the rule we employed to select our sample—only including comments containing particular word strings—did not capture all relevant comments. As a result, this may have been a convenient rather than complete sampling of those comments. Another possible reason for the limited number of data-centered comments is that discussing profile data is only one of the many uses members make of each other’s posted medical information, with one such use, posting prescripted comments, being actively encouraged by the site design. A member, with one click, can post a prescripted comment to another member, thanking him or her for entering personal information. Although we found that about half of the comments did not include this prescripted comment and were written “from scratch,” that design decision may influence how members use each other’s profiles.

At the same time, the presence and apparent value of comments that explicitly reference data suggest the need for design innovations that promote data-centered patient conversation. The current design does so by offering the ability to search for other users based on criteria including treatments, symptoms, and demographics, as well as by providing both open commenting and private messaging. Future designs could include single-click functionality to ask another user about a shared experience, enhanced visualization techniques to facilitate the interpretation of the health profile, methods to search for people based on a larger variety of characteristics, and the ability to comment on a specific portion of someone’s health profile. Our analysis also suggests that particular comments may be useful to a wider audience; therefore, a method of identifying, archiving, and presenting such comments for other individuals should be investigated.

## Abbreviations

ALS: amyotrophic lateral sclerosis
BiPAP: bilevel positive airway pressure
FRS: functional rating scale
